# Prediction of turbulence eddy dissipation of water flow in a heated metal foam tube

**DOI:** 10.1038/s41598-020-76260-6

**Published:** 2020-11-06

**Authors:** Meisam Babanezhad, Iman Behroyan, Ali Taghvaie Nakhjiri, Mashallah Rezakazemi, Azam Marjani, Saeed Shirazian

**Affiliations:** 1 Institute of Research and Development, Duy Tan University, Da Nang, 550000 Vietnam; 2 Faculty of Electrical – Electronic Engineering, Duy Tan University, Da Nang, 550000 Vietnam; 3 Mechanical and Energy Engineering Department, Shahid Beheshti University, Tehran, Iran; 4 Department of Petroleum and Chemical Engineering, Science and Research Branch, Islamic Azad University, Tehran, Iran; 5 Faculty of Chemical and Materials Engineering, Shahrood University of Technology, Shahrood, Iran; 6 Department for Management of Science and Technology Development, Ton Duc Thang University, Ho Chi Minh City, Vietnam; 7 Faculty of Applied Sciences, Ton Duc Thang University, Ho Chi Minh City, Vietnam; 8 Department of Chemical Sciences, Bernal Institute, University of Limerick, Limerick, Ireland; 9 Laboratory of Computational Modeling of Drugs, South Ural State University, 76 Lenin Prospekt, Chelyabinsk, Russia 454080

**Keywords:** Mathematics and computing, Engineering, Chemical engineering, Mechanical engineering

## Abstract

The insertion of porous metal media inside the pipes and channels has already shown a significant heat transfer enhancement by experimental and numerical studies. Porous media could make a mixing flow and small-scale eddies. Therefore, the turbulence parameters are attractive in such cases. The computational fluid dynamics (CFD) approach can predict the turbulence parameters using the turbulence models. However, the CFD is unable to find the relation of the turbulence parameters to the boundary conditions. The artificial intelligence (AI) has shown potential in combination with the CFD to build high-performance predictive models. This study is aimed to establish a new AI algorithm to capture the patterns of the CFD results by changing the system’s boundary conditions. The ant colony optimization-based fuzzy inference system (ACOFIS) method is used for the first time to reduce time and computational effort needed in the CFD simulation. This investigation is done on turbulent forced convection of water through an aluminum metal foam tube under constant wall heat flux. The ANSYS-FLUENT CFD software is used for the simulations. The *x* and *y* of the fluid nodal locations, inlet temperature, velocity, and turbulent kinetic energy (TKE) are the inputs of the ACOFIS to predict turbulence eddy dissipation (TED) as the output. The results revealed that for the best intelligence of the ACOFIS, the number of inputs, the number of ants, the number of membership functions (MFs) and the rule are 5, 10, 93 and 93, respectively. Further comparison is made with the adaptive network-based fuzzy inference system (ANFIS). The coefficient of determination for both methods was close to 1. The ANFIS showed more learning and prediction times (785 s and 10 s, respectively) than the ACOFIS (556 s and 3 s, respectively). Finding the member function versus the inputs, the value of TED is calculated without the CFD modeling. So, solving the complicated equations by the CFD is replaced with a simple correlation.

## Introduction

The transport phenomena and heat transfer within the permeable media are vital in several engineering applications such as membranes, packed-bed catalytic reactors, electronic cooling, and adsorption. Thermal management goal aims to ensure that each part’s temperature in a chemical/physical system remains within the definite operational domains^1,2^, or to guarantee the improved enforced convective heat transfer in different applications such as heat exchangers, cooling towers, and solar collectors^3,4^.

The utilization of metal froth-filled improved tubes is broadly examined in literature^5–7^. Calmidi et al.^8^, Zhao et al.^9^, and Kim et al.^10^ conducted convection heat transfer implementation of metal foam-occupied channels with various structures experimentally and numerically to understand the underlying phenomena. The system’s parameters such as pore density, metal froth porosity, and channel geometry were extensively included affecting the fluid flow as well as heat transport. Moreover, Zhao et al.^11,12^ assessed heat transfer and boiling flow in foam-filled horizontal metal tubes, where those elements affecting heat transfers and pressure drops were totally tested and studied.

Computational fluid dynamics (CFD) tools are commonly used to predict thermal and hydrodynamic parameters of convective flows^13,14^. The CFD tools prevent the extra expenses of the experiments coming from the trial and error processes. However, the CFD approach has its own expenses, especially for complex cases (e.g. two-phase flows, turbulent flows, complicated geometries, etc.). Modeling such complicated cases require a lot of computational efforts and high-performance computers. Although the CFD method predicts the fluid flow characteristics (e.g. temperature, velocity, pressure, turbulence parameters, etc.), this approach cannot find the relationship of such parameters with each other at an accepted level.

Nowadays, scientists studied the effects of artificial intelligence (AI) algorithms on evaluating fluid characteristics to avoid solving complicated CFD cases and saving computational efforts. Once the CFD results are obtained, the AI algorithm can learn the data and find the general pattern of the CFD data changes. Besides, the AI algorithm can find the relationship between the flow characteristics. In new researches, a combination of *Fuzzy Inference System* (FIS) as the main core of intelligence with new meta-heuristic algorithms such as ant colony optimization, differential evolution, genetic, and particle swarm optimization algorithms were considered to study fluid dynamics and heat transfer. Changing in FIS parameters are important for achieving the best intelligence.

Recently some studies considered the contribution of the ANFIS (adaptive network-based fuzzy inference system) with the CFD to predict fluid flow characteristics^15–17^. The studies just simply discussed the application of the ANFIS for the facilitation of the CFD. But there is no discussion about the details of the algorithm setup and the effect of using the other trainers (i.e. ant colony optimization, differential evolution, genetic, and particle swarm optimization algorithms). Besides, there are not any studies to present the ability of artificial intelligence for correlating fluid flow characteristics.

According to the above discussion, for filling the research gaps, this study tries to use the ant colony optimization-based fuzzy inference system (ACOFIS) method for the first time to help the CFD model by the reduction of the number of the simulations. The efficiency of ACOFIS is compared with the ANFIS. The heat transfer and hydrodynamics of water flow inside an aluminum metal foam tube exposed to a fixed heat transfer flux is selected as a case study. The simulation is done for several inlet velocities and inlet temperatures. The *x* and *y* of the fluid nodal locations, the temperature at the inlet (T), the velocity (V), and turbulent kinetic energy (TKE) are the inputs of the ACOFIS to predict turbulence eddy dissipation (TED) as the output. Finally, a correlation is developed for finding the values of TED as a function of *x*, *y*, T, V, and TKE.

## Methodology

### Geometry and boundary conditions

The geometry of the case study is a cylindrical tube with a diameter of 10 mm, and a length of 1 m. The fluid with uniform temperature of T_0_ and uniform velocity of u_0_ enters the pipe. It is assumed that the heat flux at the wall q_w_ is constant. The summary of the simulation cases and the boundary conditions are given in Table 1.

**Table 1:** CFD cases.

Case no.	Wall (kW/m^2^)	T_inlet_	V_inlet_ (m/s)
1	q = 95	295 k	0.91
2		305 k	
3		325 k	
4		335 k	

### CFD method

The assessment is carried out for fluid with the conditions: steady state, incompressible, 3D and turbulent regime within a tubular geometry completely occupied with porous material submerged with a one-phase Newtonian fluid. Furthermore, the introduced porous material in the tube is supposed homogenous and isotropic with a uniform permeability magnitude and porosity. The characteristics of porous media including material, porosity, permeability, and PPI are aluminum, 0.8, 5 × 10^–8^ m^2^, and 10, respectively. Moreover, temperature-caused change in the thermo-physical features of the solid matrix and the working fluid is considered negligible. Furthermore, it is assumed that viscous dissipation, natural convection gravitational effects, and radiation heat transfer will have an insignificant impact on temperature and velocity distributions as well as the solid and fluid are regarded in local thermodynamic equilibrium. The equations of mass, energy, and momentum conservations may be expressed as^18–22^:

Continuity:

1 $$\frac{\partial \rho }{{\partial t}} + \nabla \cdot \left( {\rho \overrightarrow {u} } \right) = 0$$

Momentum:

2 $${\raise0.7ex\hbox{$1$} \!\mathord{\left/ {\vphantom {1 \varepsilon }}\right.\kern-\nulldelimiterspace} \!\lower0.7ex\hbox{$\varepsilon $}}\frac{{\partial \rho \overrightarrow {u} }}{\partial t} + {\raise0.7ex\hbox{$1$} \!\mathord{\left/ {\vphantom {1 \varepsilon }}\right.\kern-\nulldelimiterspace} \!\lower0.7ex\hbox{$\varepsilon $}}\nabla \cdot \left( {\rho \overrightarrow {u} \overrightarrow {u} } \right) = - \nabla p + \varepsilon \rho \overrightarrow {g} + {\raise0.7ex\hbox{$1$} \!\mathord{\left/ {\vphantom {1 \varepsilon }}\right.\kern-\nulldelimiterspace} \!\lower0.7ex\hbox{$\varepsilon $}}\nabla \left[ {\mu_{{}}^{e} \left( {\nabla \overrightarrow {u} + \left( {\nabla \overrightarrow {u} } \right)^{T} } \right)} \right] + \vec{F}$$

*F* is given as:

3 $$\vec{F} = \left( {\frac{{\mu_{f} }}{K} + \frac{{\rho_{f} C\left| v \right|}}{\sqrt K }} \right)\vec{v}$$

where *K* is the permeability and *C* is the inertial coefficient given as^23,24^:

4 $$C = 0.00212\left( {1 - \varepsilon } \right)^{ - 0.132} \left( {\frac{{d_{l} }}{{d_{p} }}} \right)^{ - 1.63}$$

where *d*_*l*_ is the ligament and *d*_*p*_ is the pore size obtained as:

5 $$d_{l} = 1.18d_{p} \sqrt {\frac{1 - \varepsilon }{{3\pi }}} \left( {\frac{1}{{1 - e^{{{\raise0.7ex\hbox{${ - \left( {1 - \varepsilon } \right)}$} \!\mathord{\left/ {\vphantom {{ - \left( {1 - \varepsilon } \right)} {0.04}}}\right.\kern-\nulldelimiterspace} \!\lower0.7ex\hbox{${0.04}$}}}} }}} \right)$$

6 $$d_{p} = 0.0254(m)/\omega (PPI)$$

In Eq. (6), *ω* denotes pore density. Also, PPI stands for pores per inch.

Energy conservation equation:

7 $$\frac{\partial \rho H}{{\partial t}} + \nabla \cdot \left( {\rho \overrightarrow {{u_{{}} }} H} \right) = \nabla \left[ {{\text{k}}_{e} \left( {\nabla T} \right)} \right]$$

where effective thermal conductivity parameter is calculated as:

8 $$k_{e} = (1 - \varepsilon )k_{s} + \varepsilon k_{F}$$

where subscripts *s* and *F* refer to the solid and fluid, respectively.

The equations for water properties are written as^25^:

Density:

9 $$\begin{gathered} \rho_{F} = 2446 - 20.674T + 0.11576T^{2} - 3.12895 \times 10^{ - 4} T^{3} + 4.0505 \times 10^{ - 7} T^{4} - 2.0546 \times 10^{ - 10} T^{5} \hfill \\ \hfill \\ \end{gathered}$$

Viscosity:

10 $$\mu_{F} = A10^{{\left( {\frac{B}{T - C}} \right)}}$$

where A = 2.414 × 10–5, B = 247.8, and C = 140.

Specific heat:

11 $$\left( {C_{p} } \right)_{F} = \exp \left( {\frac{8.29041 - 0.012557T}{{1 - \left( {1.52373 \times 10^{ - 3} } \right)T}}} \right)$$

### Turbulence modelling

The turbulence effect is simulated by the standard k–ɛ model reported by Launder and Spalding^26,27^ as follows:

12 $$\frac{\partial }{\partial t}\left( {\rho k} \right) + \nabla \cdot (\rho k\overrightarrow {u} ) = \nabla \cdot \left[ {\left( {\frac{{\mu^{t} }}{{\sigma_{k} }}} \right)\nabla (k_{{}} )} \right] + G - \rho \varepsilon$$

13 $$\frac{\partial }{\partial t}\left( {\rho_{{}} \varepsilon_{{}} } \right) + \nabla \cdot (\rho \varepsilon \overrightarrow {u} ) = \nabla \cdot \left[ {\frac{{\mu_{{}}^{t} }}{{\sigma_{\varepsilon } }}\nabla \varepsilon } \right] + \frac{\varepsilon }{k}(C_{1\varepsilon } G_{k} - C_{2\varepsilon } \rho_{f} \varepsilon )$$

14 $$G_{{}} = \mu_{t} (\nabla \overrightarrow {{u_{{}} }} + (\nabla \overrightarrow {{u_{{}} }} )^{T} )$$

15 $$\mu_{t} = \rho_{{}} C_{\mu } \frac{{k^{2} }}{\varepsilon }$$

$$C_{\mu } = 0.09,\sigma_{k} = 1.00,\sigma_{\varepsilon } = 1.30,C_{1\varepsilon } = 1.44,C_{2\varepsilon } = 1.92$$

where *G* is the production of turbulence due to the liquid shear stress.

### Numerical methods

All numerical methods, using in this study are available in the CFD package of ANSYS-Fluent. Fluent is finite volume (FV) scheme for converting the partial differential equations (PDEs) into algebraic equations for numerical solutions. Second-order upwind scheme is adopted for discretization of the momentum, energy, TKE and turbulent dissipation rate equations. *SIMPLE* algorithm is employed for the pressure–velocity coupling scheme. ANSYS Design Modeler is used for meshing process. The discretization grid is included 250, 10 and 50 nodes in the axial, radial and circumferential directions, respectively.

### Ant colony optimization (ACO)

The artificial agents are used by the generic ACO algorithm cooperating to discover the decent solutions of continuous/discrete optimization tasks. In this optimization approach, the agents are termed ants mimicking searching performance of biological systems in discovering the shortest pathway to reach the source of food in nature. AS (Ant system)^28^ is used for the *Traveling Salesman Problem* (TSP), in which the ants make solutions via an iterative manner, and produce a certain quantity of pheromone over the traveled pathways. Selecting the path is a stochastic process in terms of two parameters of the heuristic and pheromone values for a product. When ant *k* presently place at *i* city for TSP, selects city *j* with a probability as^29^:

16 $${P}_{il}^{k}=\frac{{[{\tau }_{ij}]}^{\alpha }{[\upeta ]}^{\beta }}{\sum l\in {N}_{i}^{k}{[{\tau }_{il}]}^{\alpha }{[{\upeta }_{il}]}^{\beta }}\quad if\quad j\in {N}_{i}^{k}$$

In Eq. (16) α and β denote 2 modification parameters determining the relative significance between the heuristic in formation ${\upeta}_{ij}$, the pheromone trail ${\tau }_{ij}$, and ${N}_{i}^{k}$ is the asset of city contestants not met by the ant *k*. The pheromone value indicates the number of ants choosing the trail presently; however, the heuristic value is a problem-based quality action. Reaching a decision point, it probably selects the trail with the greater heuristic and pheromone values. When the ant reaches the destination, the equivalent solution to the path passed by the ant is assessed and the equivalent pheromone value on the pathway is updated as^29^:

17 $${\tau }_{ij}\left(m+1\right)=\rho {\tau }_{ij}\left(m\right)+\sum_{k=1}^{\stackrel{\sim }{N}}\Delta {\tau }_{ij}^{k}(m)$$

where $\rho$ represents the pheromone trail perseverance and $\Delta {\tau }_{ij}^{k}(m)$ shows the quantity of pheromone deposited by the ant *k* on the traversed arc *ij*. Stutzle et al.^30^ indicated that the enhanced behavior could be attained by robust use of the best solutions, along with an operative mechanism to avoid initial search stagnation. It should be noted that despite numerous changes in the initial ant system to various problems, only fewer related reports^31^ exist on using ACO for estimating the parameters as detailed in this work.

### Fuzzy inference system (FIS)

Fuzzy inference system (FIS) is a common computing framework in terms of the fuzzy set theory concepts, fuzzy reasoning, and fuzzy if–then rules. It was successfully utilized in fields like automatic control, data classification, computer vision, expert systems, and decision analysis. Three various kinds of fuzzy reasoning exist implementing Sugeno and Takagi if–then rules in FIS structure^32^. In this study, x- and y-direction, inlet temperature, turbulence kinetic energy (TKE) and velocity (V) are considered to acquire TED as output. The signals incoming are multiplied based on the AND rule. For instance, the ith rule’s function is^33^:

18 $${w}_{i}={\mu }_{Ai}\left(X\right) {\mu }_{Bi}\left(Y\right){\mu }_{Ci}(Tin){\mu }_{Di}(\mathrm{TKE}) {\mu }_{Ei}(\mathrm{V})$$

where ${w}_{i}$ represent outcoming signal and ${\mu }_{Ai}$, ${\mu }_{Bi}$, ${\mu }_{Ci}$, ${\mu }_{Di}$ and ${\mu }_{Ei}$ denote the signals incoming from MFs run over the inputs x-direction (X), y-direction (Y), inlet temperature (T_in_), TKE and velocity (V).

The relative value of each rule’s firing strength is obtained equal to the weight over the overall quantity of the firing strengths of all rules^33^:

19 $$\stackrel{-}{{w}_{i}}=\frac{{w}_{i}}{\sum \left({w}_{i}\right)}$$

where $\stackrel{-}{{w}_{i}}$ denotes the normalized firing strength. The defuzzication step used the function of a consequence if–then rule provided by Takagi and Sugeno^32^.

Hence, the node function is^33^:

20 $$\stackrel{-}{{w}_{i}}{f}_{i}=\stackrel{-}{{w}_{i}}({p}_{i}X+{q}_{i}Y+{r}_{i}Tin+{s}_{i}\mathrm{TKE}+{t}_{i}V+{u}_{i})$$

where ${p}_{i}$, ${q}_{i}$, ${r}_{i}$, ${s}_{i}$, ${t}_{i}$, and ${u}_{i}$ denote the parameters of if–then rules.

## Results and discussion

**Figure 1:**
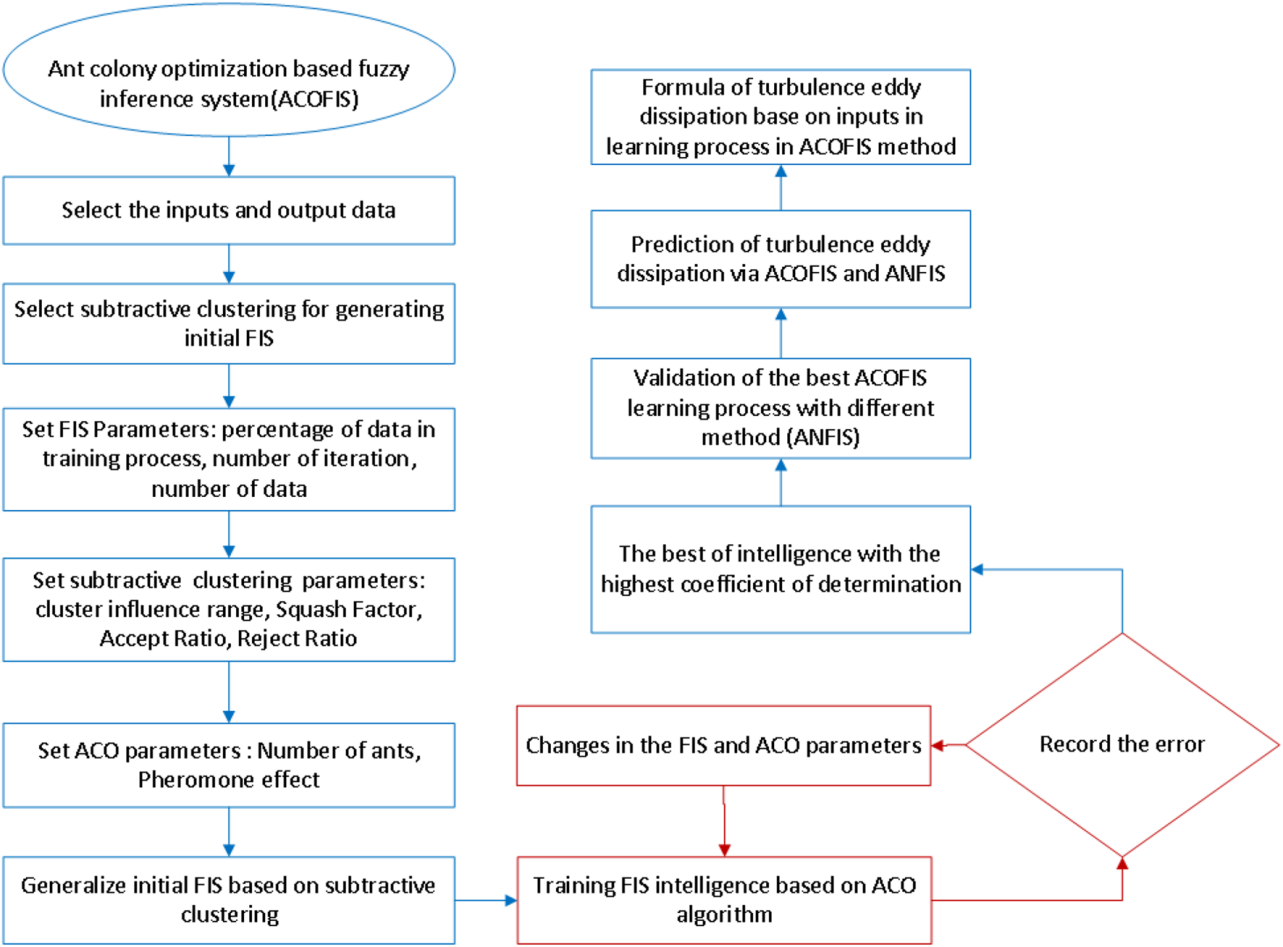
Flowchart of ACOFIS method.

Figure 1 illustrates different steps of the fuzzy inference system (FIS) using the ant colony optimization (ACO) algorithm for learning the CFD results. Primarily, the inputs and the output of the ACOFIS are defined. The subtractive clustering is selected as the type of data clustering and for the inertia FIS. According to the CFD results, 2148 data are available for learning. The ACOFIS trains 70% of the CFD data for 60 iterations. The subtractive clustering parameters including the cluster influence range, the squash factor, the accept ratio, and the reject ratio are determined. For the ACO parameters, the number of ants and the pheromone effect are 10 and 0.2, respectively. Then the training process of the FIS is done based on the ACO algorithm. The TED predicted by the ACOFIS is compared with that predicted by the CFD. All errors of the ACOFIS predictions are calculated based on the CFD results. It should be noted that the values of ACO and FIS parameters are adjusted until the best intelligence is obtained. The sensitivity test for this adjustment is not considered in this study. The best intelligence of the ACOFIS is related to the highest coefficient of determination. Further validation is done by a comparison of the ACOFIS results with another artificial intelligence algorithm. This algorithm is also considered in this study for more comparison. In the end, the relation of the TED to the TKE and the inlet boundary conditions (velocity and temperature) is obtained. So, solving the complicated equations by the CFD is replaced with a simple correlation.

Table 2 summarized all parameters that have been adopted for the setup of ANFIS and ACOFIS parameters. According to Table 2, the values of all parameters are equal for both methods. The type of FIS, membership function, and clustering are also similar for both of them.

**Table 2:** ACOFIS and ANFIS initial parameters.

Methods	ACOFIS	ANFIS
Number of inputs	5	5
Maximum of Iteration	60	60
Percentage of data in learning processes	70	70
Programming software	Matlab	Matlab
Type of FIS	Sugeno	Sugeno
Clustering type	Subtractive clustering	Subtractive clustering
Type of membership functions	Gaussmf	Gaussmf
Number of input membership function	94	111
Number of rules as FIS parameter	94	111
Cluster influence range as a subtractive clustering parameter	0.2	0.2
Squash factor as a subtractive clustering parameter	1.25	1.25
Accept ratio as a subtractive clustering parameter	0.5	0.5
Reject ratio as a subtractive clustering parameter	0.15	0.15
Number of ants as a ACO parameter	10	–
Pheromone effect as a ACO parameter	0.2	–

**Figure 2:**
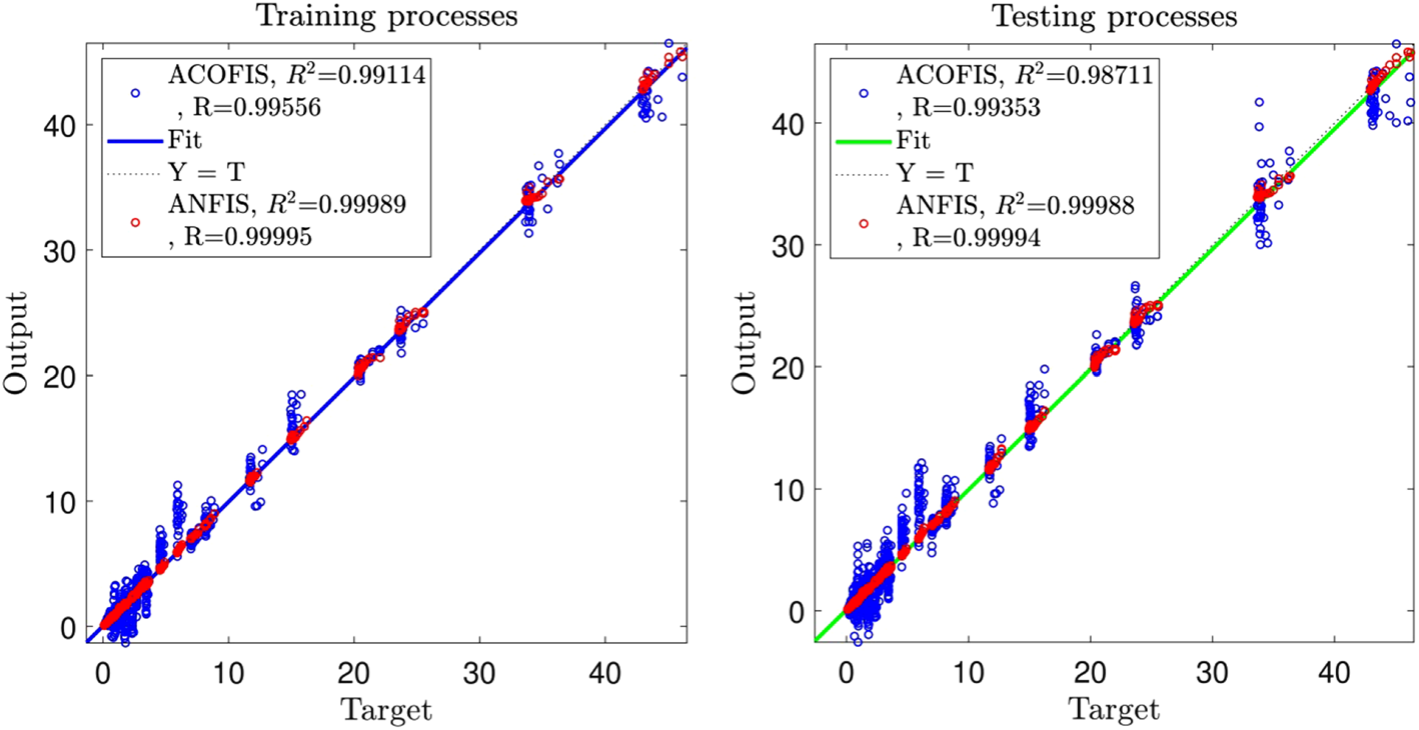
Regression plot of training and testing processes for the best FIS trained by ant colony optimization algorithm and the best result of ANFIS.

**Figure 3:**
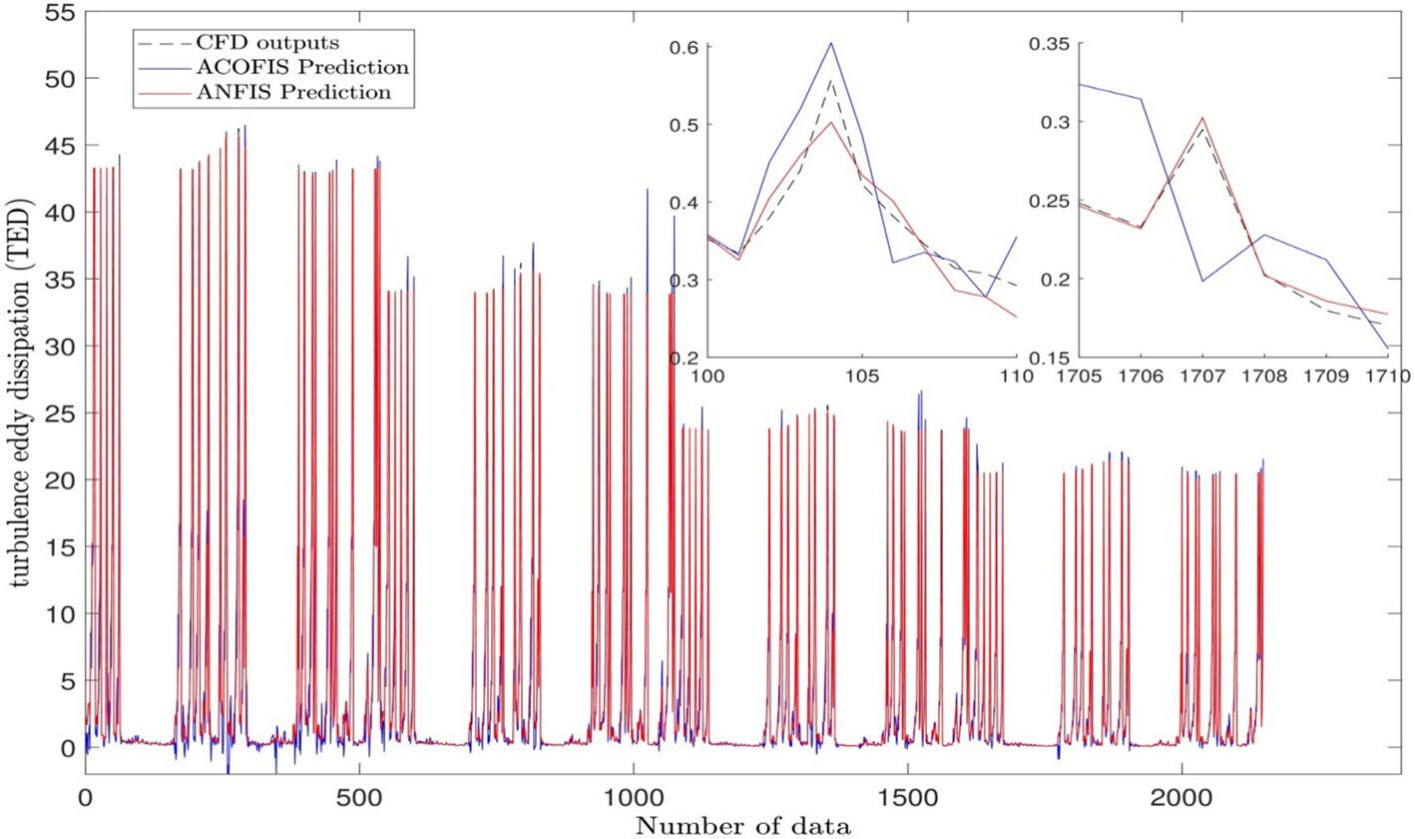
Validation of ANFIS and ACOFIS prediction for prediction of turbulence eddy dissipation in each node.

As shown in Fig. 2, the values of the correlation coefficient (R) and the coefficient of determination (R^2^) of the ANFIS (R = 0.99994 and R^2^ = 0.99988) are a little higher than those of the ACOFIS (R = 0.99353 and R^2^ = 0.98711). Figure 3 shows the prediction of the TED of both ANFIS and ACOFIS benchmarking the CFD results. Magnifying the graph, the ANFIS prediction shows a little more compatible results with the CFD than the ACOFIS. Table 3 shows a better comparison between the ANFIS and the ACOFIS. Based on the information of this table, for roughly the same values of R and R^2^ for both methods, the ANFIS takes more learning and prediction times (785 s and 10 s respectively) than the ACOFIS (556 s and 3 s respectively).

**Table 3:** ACOFIS and ANFIS different errors and learning and prediction times.

Methods	ACOFIS	ANFIS
Training correlation coefficient (R)	0.995558913	0.999947369
Training coefficient of determination (R^2^)	0.99113755	0.99989474
Testing correlation coefficient (R)	0.993532369	0.999941344
Testing coefficient of determination (R^2^)	0.987106569	0.999882692
Learning time (s)	555.8053779	785.4094361
Prediction time (s)	2.8730278	10.402829

**Figure 4:**
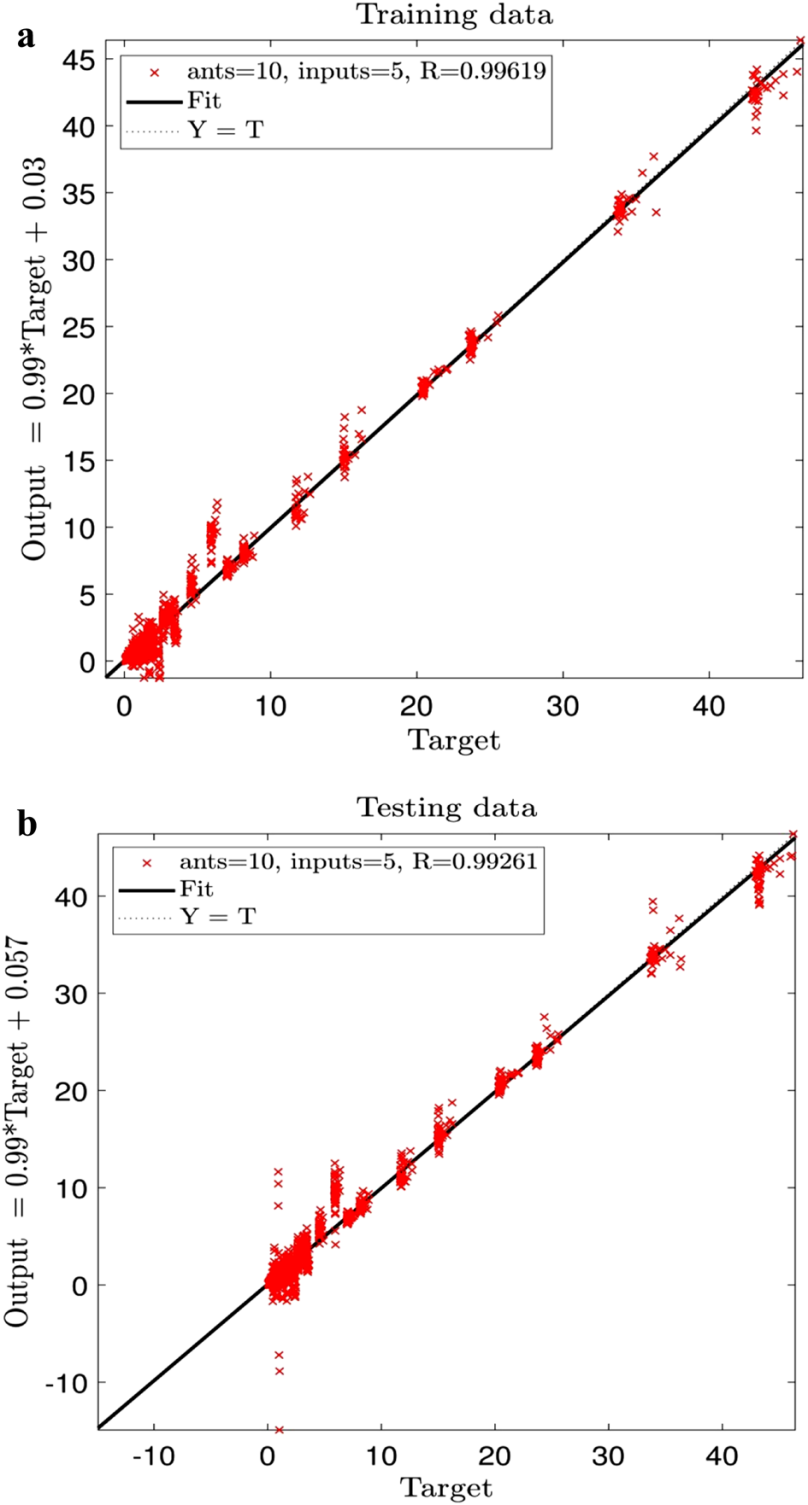
(**a**) Training process of ACOFIS using five inputs when number of ants is 10 and pheromone effect is 0.2. (**b**) Testing process of ACOFIS using five inputs when number of ants is 10 and pheromone effect is 0.2.

The integration of the ACOFIS artificial intelligence with the CFD modeling in prediction of heat transfer and hydrodynamics of water flow through an Aluminum metal foam tube is investigated. The CFD modeling is done by ANSYS-FLUENT CFD software. The ACOFIS method uses the *x* and *y* of the fluid nodal locations, the temperature at the inlet (T), the velocity (V) and TKE as the inputs to predict TED as the output. Figure 4a,b show that the best intelligence could be obtained if all five inputs are included. Besides, the number of ants, and the number of membership functions (MFs) and the rule should be 10 and 93, respectively. According to Fig. 4, the regression number is roughly equal to 1 for both training and testing.

**Figure 5:**
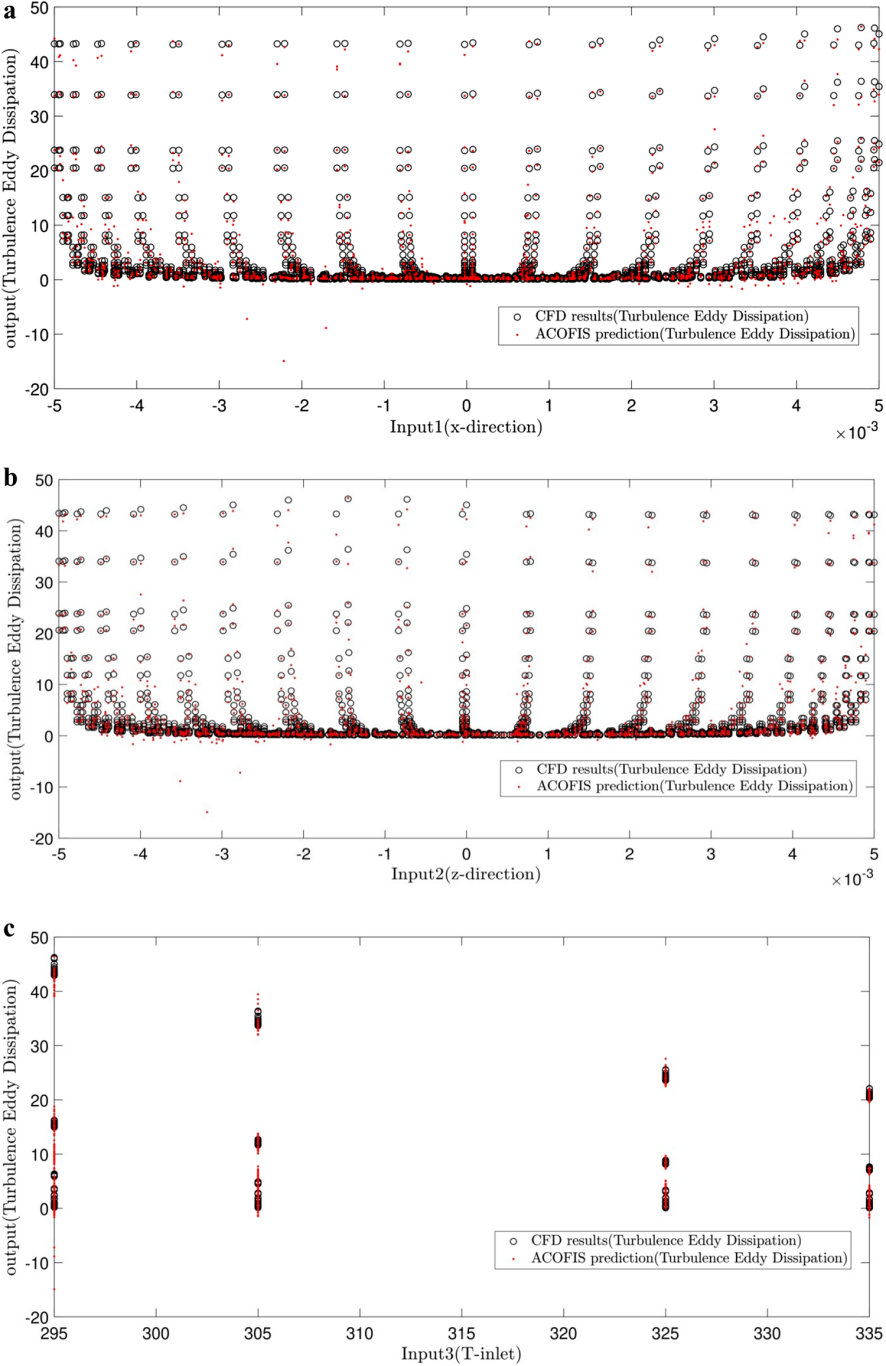

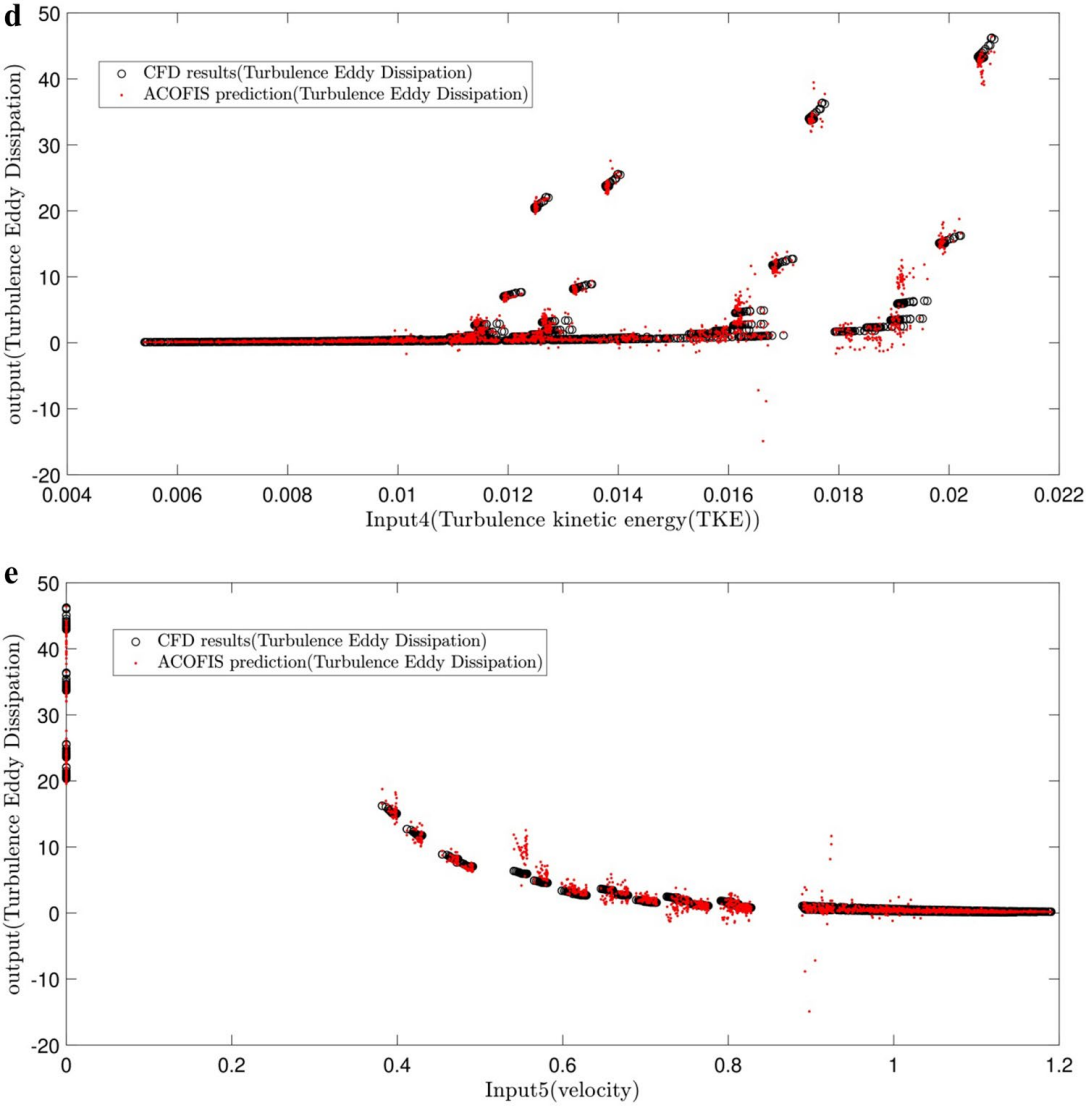
Comparison of ACOFIS output with CFD data (turbulence eddy dissipation) based on (**a**) x-direction as first input of ACOFIS, (**b**) y-direction as second input of ACOFIS, (**c**) inlet temperature as third input of ACOFIS, (**d**) Turbulence kinetic energy (TKE) as fourth input of ACOFIS, (**e**) velocity (V) as fifth input of ACOFIS.

A comparative study is done between the CFD and the ACOFIS prediction of TED (shown in Fig. 5a–e). The results revealed that the predicted TED by the ACOFIS is in a good agreement with those by the CFD model for all 5 inputs.

**Figure 6:**
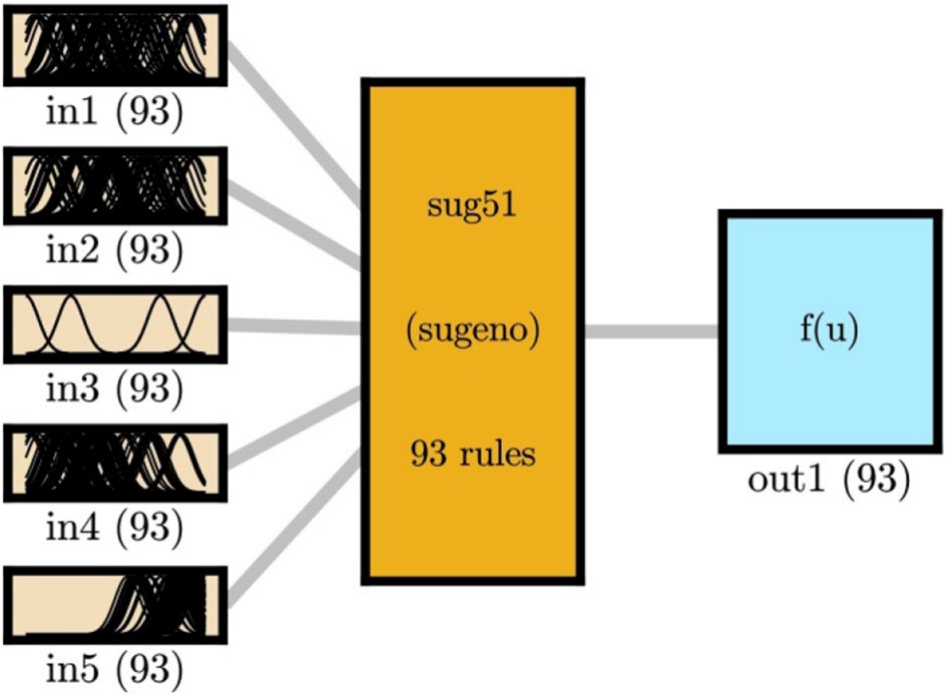
FIS structure trained by ACO algorithm as trainer in the highest level of intelligence.

The schematic diagram of the data clustering is illustrated by Fig. 6. The number of MFs for all inputs and the output, and the number of rules is equal to 93.

**Figure 7:**
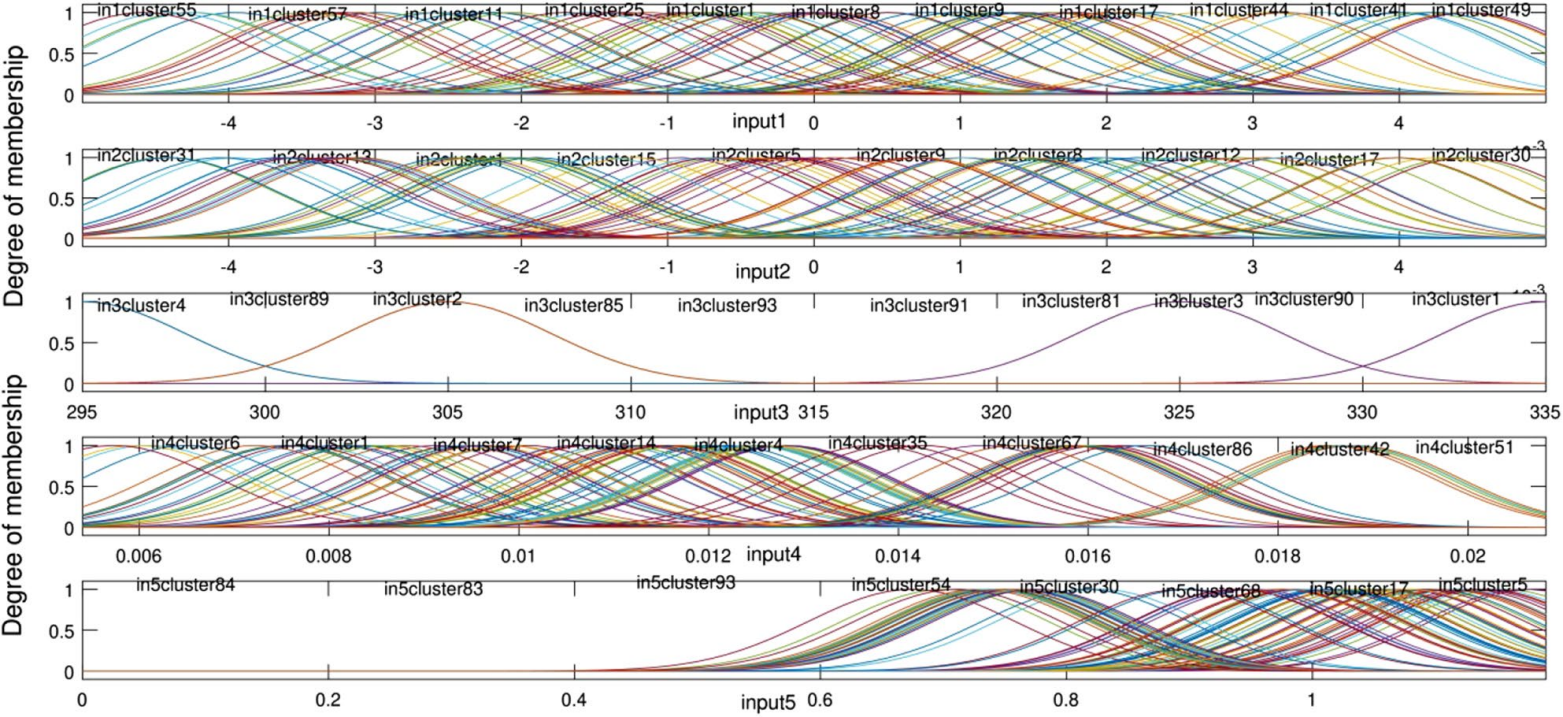
Degree of membership in the highest level of intelligence.

Figure 7 shows the degree of MFs for all inputs and all number of clustering. According to Fig. 7, the value of the Gaussian membership function can be determined for each value of the input. It should also be noted that there is a domain for all inputs. For example, the *x* and *y* are between ± 5 cm. The temperature limitation is from 295 to 335 K. The velocity must be selected from 0.006 to 0.02 m/s. The TKE is from 0 to 1 m^2^/s^2^.

21 $$TED=\frac{\sum_{i=1}^{93}\sum_{j=1}^{93}\sum_{k=1}^{93}\sum_{l=1}^{93}\sum_{n=1}^{93}\left({\mu }_{1i}\times {\mu }_{2j}\times {\mu }_{3k}\times {\mu }_{4l}\times {\mu }_{5n}\right)\times \left({o}_{m}X\times {p}_{m}Y\times {q}_{m}{T}_{inlet}\times {r}_{m}TKE\times {s}_{m}V\times {z}_{m}\right)}{\sum_{i=1}^{93}\sum_{j=1}^{93}\sum_{k=1}^{93}\sum_{l=1}^{93}\sum_{n=1}^{93}\left({\mu }_{1i}\times {\mu }_{2j}\times {\mu }_{3k}\times {\mu }_{4l}\times {\mu }_{5n}\right)}$$

in which

$${\mu }_{1i}={e}^{\frac{-{\left(x-{c}_{i}\right)}^{2}}{2{\sigma }^{2}}}, {\mu }_{2j}={e}^{\frac{-{\left(x-{c}_{j}\right)}^{2}}{2{\sigma }^{2}}}, {\mu 3k=e}^{\frac{-{\left(x-{c}_{k}\right)}^{2}}{2{\sigma }^{2}}}, \mu 4l={e}^{\frac{-{\left(x-{c}_{l}\right)}^{2}}{2{\sigma }^{2}}}, {\text{and}} \mu 5n={e}^{\frac{-{\left(x-{c}_{n}\right)}^{2}}{2{\sigma }^{2}}}$$

According to Eq. (21), turbulence eddy dissipation (TED) as the output is correlated to the *x* and *y* of the fluid nodal locations, the temperature at the inlet (T), the velocity (V), and TKE as the inputs. Table 4 shows the Gaussian function and its parameters.

**Table 4:** Gaussian membership function equation in ACOFIS learning process.

Membership function	Equation
Gaussian	${e}^{\frac{-{\left(x-c\right)}^{2}}{2{\sigma }^{2}}}$

Table S1 (Supplementary Information) illustrates the values of *C* and σ as the parameters of Gaussian function at each cluster and for the best intelligence condition. Table S2 (Supplementary Information) illustrates consequent parameters (p, q, r, s, t, and u) at each cluster. After the determination of the value of the Gaussian membership function (µ), the value of TED is calculated by Eq. (21) without using the CFD. This leads to a lot of reduction in time and computational effort.

## Conclusion

This study was aimed to offer a straightforward way for the reduction of the number of CFD simulations. This could be useful for complex cases like 3D geometries or turbulent flows. So, the 3D modeling of turbulent forced convection of water in a pipe was considered. In addition, the CFD approach is unable to find the relationship of the fluid flow parameters (i.e., velocity, temperature, turbulence parameters, etc.) with each other. This investigation aims to show the contribution of the ACOFIS artificial intelligence method, for the first time, in the prediction of heat transfer and hydrodynamics of water flow inside a metal foam tube. The ACOFIS was integrated with the CFD modeling. Once the CFD results are obtained, the ACOFIS can learn the data and find the general pattern of the CFD data changes.

The ANSYS-FLUENT CFD software was used for simulation of forced convection of water inside the metal foam tube. The water enters the tube by different inlet temperatures (i.e. 295, 305, 325 and 335 K). The *x* and *y* of the fluid nodal locations, the temperature at the inlet (T), the velocity (V), and turbulent kinetic energy (TKE) were considered as the inputs. The turbulence eddy dissipation (TED) was selected as the output. All the predictions were done on a cross-section in 0.3 m of the tube length. For more comparison, the efficiency of ACOFIS is compared with the adaptive network-based fuzzy inference system (ANFIS). The following results can be found from this investigation:

- The best intelligence (i.e. R ~ 1) could be obtained for the number of inputs equal to five. In addition, the number of ants, and the number of membership functions (MFs) and the rule should be 10 and 93, respectively.
- The comparative study between the CFD and the ACOFIS prediction of TED revealed that the predicted TED by the ACOFIS is in a good agreement with those by the CFD.
- The function of the degree of MFs versus all inputs and numbers of clustering was graphically determined. Once the values of the inputs are determined, the value of TED can be calculated without the CFD modeling. So, solving the complicated equations by the CFD could be replaced with a simple correlation.
- The coefficient of determination and the correlation of coefficient for both methods of ANFIS and ACOFIS were close to 1 at the best intelligence. The ANFIS showed more learning and prediction times (785 s and 10 s, respectively) than the ACOFIS (556 s and 3 s, respectively).

## Supplementary information

Supplementary Information.
