# Supplementary material for: Prediction of turbulence eddy dissipation of water flow in a heated metal foam tube
Source: Sci Rep. 2020 Nov 6;10:19280. doi: 10.1038/s41598-020-76260-6 (PMC7648062; doi:10.1038/s41598-020-76260-6)
Supplement: Supplementary file 1 — Supplementary Information. [file 41598_2020_76260_MOESM1_ESM.docx]

**(Supplementary file)**

**Prediction of turbulence eddy dissipation of water flow in a heated metal foam tube**

Meisam Babanezhad^1,2^, Iman Behroyan^3^, Ali Taghvaie Nakhjiri^4^, Mashallah Rezakazemi^5^, Azam Marjani^6,7,*^, Saeed Shirazian^8,9^

^1^Institute of Research and Development, Duy Tan University, Da Nang 550000, Vietnam

^2^Faculty of Electrical – Electronic Engineering, Duy Tan University, Da Nang 550000, Vietnam

^3^ Mechanical and Energy Engineering Department, Shahid Beheshti University, Tehran, Iran

^4^ Department of Petroleum and Chemical Engineering, Science and Research Branch, Islamic Azad University, Tehran, Iran

^5^ Faculty of Chemical and Materials Engineering, Shahrood University of Technology, Shahrood, Iran

^6^ Department for Management of Science and Technology Development, Ton Duc Thang University, Ho Chi Minh City, Vietnam

^7^ Faculty of Applied Sciences, Ton Duc Thang University, Ho Chi Minh City, Vietnam

^8^ Department of Chemical Sciences, Bernal Institute, University of Limerick, Limerick, Ireland

^9^ Laboratory of Computational Modeling of Drugs, South Ural State University, 76 Lenin prospekt, 454080 Chelyabinsk, Russia

^*^Corresponding author, E-mail: azam.marjani@tdtu.edu.vn

Table S1: Inputs membership functions parameters in ACOFIS intelligence learning process.

| **Number of cluster** | **Type of MFs** | **σ** | ***C*** |  | **Number of cluster** | **Type of MFs** | **σ** | ***C*** |
| --- | --- | --- | --- | --- | --- | --- | --- | --- |
| 'in5cluster1' | 'gaussmf' | 0.0841272 | 1.0394214 |  | 'in5cluster47' | 'gaussmf' | 0.0841272 | 1.0304538 |
| 'in5cluster2' | 'gaussmf' | 0.0841272 | 1.0760247 |  | 'in5cluster48' | 'gaussmf' | 0.0841272 | 1.1180614 |
| 'in5cluster3' | 'gaussmf' | 0.0841272 | 1.0596087 |  | 'in5cluster49' | 'gaussmf' | 0.0841272 | 0.7384448 |
| 'in5cluster4' | 'gaussmf' | 0.0841272 | 1.077674 |  | 'in5cluster50' | 'gaussmf' | 0.0841272 | 1.0169369 |
| 'in5cluster5' | 'gaussmf' | 0.0841272 | 1.1545223 |  | 'in5cluster51' | 'gaussmf' | 0.0841272 | 0.7344121 |
| 'in5cluster6' | 'gaussmf' | 0.0841272 | 1.1443889 |  | 'in5cluster52' | 'gaussmf' | 0.0841272 | 1.0288444 |
| 'in5cluster7' | 'gaussmf' | 0.0841272 | 1.1796453 |  | 'in5cluster53' | 'gaussmf' | 0.0841272 | 0.9448789 |
| 'in5cluster8' | 'gaussmf' | 0.0841272 | 1.0979366 |  | 'in5cluster54' | 'gaussmf' | 0.0841272 | 0.7012871 |
| 'in5cluster9' | gaussmf' | 0.0841272 | 1.1370786 |  | 'in5cluster55' | 'gaussmf' | 0.0841272 | 0.7497154 |
| 'in5cluster10' | 'gaussmf' | 0.0841272 | 1.1174007 |  | 'in5cluster56' | 'gaussmf' | 0.0841272 | 0.9762549 |
| 'in5cluster11' | 'gaussmf' | 0.0841272 | 1.0570127 |  | 'in5cluster57' | 'gaussmf' | 0.0841272 | 0.7720773 |
| 'in5cluster12' | 'gaussmf' | 0.0841272 | 1.0943078 |  | 'in5cluster58' | 'gaussmf' | 0.0841272 | 0.7669395 |
| 'in5cluster13' | 'gaussmf' | 0.0841272 | 0.9910052 |  | 'in5cluster59' | 'gaussmf' | 0.0841272 | 0.7352492 |
| 'in5cluster14' | 'gaussmf' | 0.0841272 | 1.115219 |  | 'in5cluster60' | 'gaussmf' | 0.0841272 | 0.9388368 |
| 'in5cluster15' | 'gaussmf' | 0.0841272 | 1.1111082 |  | 'in5cluster61' | 'gaussmf' | 0.0841272 | 1.0524973 |
| 'in5cluster16' | 'gaussmf' | 0.0841272 | 1.1107664 |  | 'in5cluster62' | 'gaussmf' | 0.0841272 | 0.7633006 |
| 'in5cluster17' | 'gaussmf' | 0.0841272 | 0.9455463 |  | 'in5cluster63' | 'gaussmf' | 0.0841272 | 0.6741871 |
| 'in5cluster18' | 'gaussmf' | 0.0841272 | 1.0731729 |  | 'in5cluster64' | 'gaussmf' | 0.0841272 | 1.013904 |
| 'in5cluster19' | 'gaussmf' | 0.0841272 | 0.9868677 |  | 'in5cluster65' | 'gaussmf' | 0.0841272 | 0.7384003 |
| 'in5cluster20' | 'gaussmf' | 0.0841272 | 1.04594 |  | 'in5cluster66' | 'gaussmf' | 0.0841272 | 0.9934635 |
| 'in5cluster21' | 'gaussmf' | 0.0841272 | 1.1393927 |  | 'in5cluster67' | 'gaussmf' | 0.0841272 | 0.9913798 |
| 'in5cluster22' | 'gaussmf' | 0.0841272 | 1.054381 |  | 'in5cluster68' | 'gaussmf' | 0.0841272 | 0.8268881 |
| 'in5cluster23' | 'gaussmf' | 0.0841272 | 1.1071767 |  | 'in5cluster69' | 'gaussmf' | 0.0841272 | 0.8268644 |
| 'in5cluster24' | 'gaussmf' | 0.0841272 | 1.1185859 |  | 'in5cluster70' | 'gaussmf' | 0.0841272 | 1.0888802 |
| 'in5cluster25' | 'gaussmf' | 0.0841272 | 1.1327288 |  | 'in5cluster71' | 'gaussmf' | 0.0841272 | 0.8153011 |
| 'in5cluster26' | 'gaussmf' | 0.0841272 | 1.0167413 |  | 'in5cluster72' | 'gaussmf' | 0.0841272 | 0.7667317 |
| 'in5cluster27' | 'gaussmf' | 0.0841272 | 0.9465637 |  | 'in5cluster73' | 'gaussmf' | 0.0841272 | 0.7492754 |
| 'in5cluster28' | 'gaussmf' | 0.0841272 | 1.1265296 |  | 'in5cluster74' | 'gaussmf' | 0.0841272 | 0.759765 |
| 'in5cluster29' | 'gaussmf' | 0.0841272 | 1.019019 |  | 'in5cluster75' | 'gaussmf' | 0.0841272 | 0.7367413 |
| 'in5cluster30' | 'gaussmf' | 0.0841272 | 0.7758154 |  | 'in5cluster76' | 'gaussmf' | 0.0841272 | 0.7663418 |
| 'in5cluster31' | 'gaussmf' | 0.0841272 | 0.7691909 |  | 'in5cluster77' | 'gaussmf' | 0.0841272 | 0.9372603 |
| 'in5cluster32' | 'gaussmf' | 0.0841272 | 0.9736319 |  | 'in5cluster78' | 'gaussmf' | 0.0841272 | 0.7443811 |
| 'in5cluster33' | 'gaussmf' | 0.0841272 | 0.7677044 |  | 'in5cluster79' | 'gaussmf' | 0.0841272 | 0.7292367 |
| 'in5cluster34' | 'gaussmf' | 0.0841272 | 0.7676016 |  | 'in5cluster80' | 'gaussmf' | 0.0841272 | 0.7689583 |
| 'in5cluster35' | 'gaussmf' | 0.0841272 | 1.0273705 |  | 'in5cluster81' | 'gaussmf' | 0.0841272 | 0.9522573 |
| 'in5cluster36' | 'gaussmf' | 0.0841272 | 1.0093051 |  | 'in5cluster82' | 'gaussmf' | 0.0841272 | 0.7390826 |
| 'in5cluster37' | 'gaussmf' | 0.0841272 | 0.9895234 |  | 'in5cluster83' | 'gaussmf' | 0.0841272 | 0.7357504 |
| 'in5cluster38' | 'gaussmf' | 0.0841272 | 0.7686651 |  | 'in5cluster84' | 'gaussmf' | 0.0841272 | 0.711473 |
| 'in5cluster39' | 'gaussmf' | 0.0841272 | 1.073558 |  | 'in5cluster85' | 'gaussmf' | 0.0841272 | 0.7501688 |
| 'in5cluster40' | 'gaussmf' | 0.0841272 | 0.761956 |  | 'in5cluster86' | 'gaussmf' | 0.0841272 | 0.9728801 |
| 'in5cluster41' | 'gaussmf' | 0.0841272 | 0.7676227 |  | 'in5cluster87' | 'gaussmf' | 0.0841272 | 1.0778893 |
| 'in5cluster42' | 'gaussmf' | 0.0841272 | 0.7517614 |  | 'in5cluster88' | 'gaussmf' | 0.0841272 | 0.7753242 |
| 'in5cluster43' | 'gaussmf' | 0.0841272 | 0.7576848 |  | 'in5cluster89' | 'gaussmf' | 0.0841272 | 0.751109 |
| 'in5cluster44' | 'gaussmf' | 0.0841272 | 0.7421219 |  | 'in5cluster90' | 'gaussmf' | 0.0841272 | 0.9977639 |
| 'in5cluster45' | 'gaussmf' | 0.0841272 | 1.0402752 |  | 'in5cluster91' | 'gaussmf' | 0.0841272 | 0.8939339 |
| 'in5cluster46' | 'gaussmf' | 0.0841272 | 1.1728581 |  | 'in5cluster92' | 'gaussmf' | 0.0841272 | 0.8993846 |
|  |  |  |  |  | 'in5cluster93' | 'gaussmf' | 0.0841272 | 0.7512597 |

Table S2: ACOFIS method consequent parameters for predicting Turbulence Eddy Dissipation.

| **Output MFs** | **Output MFs Type** | **p** | **q** | **r** | **s** | **t** | **u** |
| --- | --- | --- | --- | --- | --- | --- | --- |
| 'out1cluster1' | 'linear' | -510.5079683 | -16024.8876 | -868.2216459 | -15514.90159 | -97.20791596 | 290998.8606 |
| 'out1cluster2' | 'linear' | -207.1167647 | -317.8657213 | -20.77329918 | 639.8928188 | 16.63138622 | 6310.63518 |
| 'out1cluster3' | 'linear' | 232.2915634 | 5256.175626 | 2471.999506 | 4160.079758 | 2.935190253 | -803440.1434 |
| 'out1cluster4' | 'linear' | -3933.304804 | -10804.84778 | -57.26815595 | -2100.330524 | 53.9965328 | 16833.48646 |
| 'out1cluster5' | 'linear' | -567.783102 | -123.6606496 | -32.90751162 | -1059.398027 | -16.95807783 | 10064.33542 |
| 'out1cluster6' | 'linear' | 152.2595159 | -27.04779832 | 41.77648138 | -984.0571592 | -33.84698884 | -13532.60101 |
| 'out1cluster7' | 'linear' | 115.8861936 | -297.5039723 | 46.69789673 | 2009.431947 | 71.4095042 | -13879.32145 |
| 'out1cluster8' | 'linear' | -1.09E+02 | -1.24E+02 | -3.329257497 | 1542.963485 | 43.51541052 | 1057.690383 |
| 'out1cluster9' | 'linear' | 52.37172812 | 5.564598175 | 12.77450935 | -9058.055161 | -294.7903133 | -3481.1792 |
| 'out1cluster10' | 'linear' | -266.9549905 | -18.44469096 | 12.66263993 | 4925.671755 | 143.2832777 | -4431.362474 |
| 'out1cluster11' | 'linear' | -2337.052443 | -1704.808088 | 6.668520122 | -895.6788993 | 24.89106116 | -2261.034384 |
| 'out1cluster12' | 'linear' | -431.2703502 | -875.860752 | 10.6674682 | 2859.293509 | 51.79575113 | -3543.182182 |
| 'out1cluster13' | 'linear' | 143.7541969 | 478.808587 | -13.0271379 | 7420.394214 | 117.5269894 | 4045.628215 |
| 'out1cluster14' | 'linear' | 125.7885925 | 68.09679238 | 3.100116449 | 1627.436946 | 50.7605154 | -989.7234126 |
| 'out1cluster15' | 'linear' | 255.0543809 | 48.91233044 | 11.82076168 | 7559.048585 | 232.7684332 | -3834.068799 |
| 'out1cluster16' | 'linear' | -291.0946155 | -818.0339032 | 6.754814408 | -3640.10178 | -116.3590667 | -1894.154115 |
| 'out1cluster17' | 'linear' | -2998.179249 | -5283.926208 | 57.67332779 | -2837.126326 | -92.02507374 | -19184.48238 |
| 'out1cluster18' | 'linear' | 56.31108118 | -117.3486669 | 69.6088518 | 212.0140299 | 7.208573792 | -22632.07704 |
| 'out1cluster19' | 'linear' | 16.62629817 | 2362.133891 | -281.9942727 | 5551.419795 | 98.12634257 | 85848.78332 |
| 'out1cluster20' | 'linear' | 2039.639547 | -2196.230854 | -34.37165611 | 10394.42567 | 382.4750639 | 11018.65899 |
| 'out1cluster21' | 'linear' | 837.1177042 | 341.3675692 | 20.17161165 | -1468.275751 | -41.70732493 | -5888.435998 |
| 'out1cluster22' | 'linear' | 390.7163939 | 288.1667843 | 1.646455034 | -1424.388609 | -30.97855497 | -509.115403 |
| 'out1cluster23' | 'linear' | -467.3518672 | -165.5642275 | 20.1221854 | -4202.065139 | -126.2394062 | -6368.653342 |
| 'out1cluster24' | 'linear' | 918.8520564 | -683.2955176 | -19.03988517 | 2521.8701 | 101.2710973 | 5668.313339 |
| 'out1cluster25' | 'linear' | 217.811346 | -485.7982238 | -59.4030763 | -4220.90622 | -122.8870115 | 17711.17083 |
| 'out1cluster26' | 'linear' | -185.1103925 | 1749.617652 | 12.03169447 | -3941.512846 | -56.3926408 | -3945.164312 |
| 'out1cluster27' | 'linear' | -2057.936016 | -2373.282459 | 0.804996165 | 6815.89569 | 132.2035565 | -469.5000502 |
| 'out1cluster28' | 'linear' | -895.4233314 | 15.75956435 | -10.33899955 | -3999.574181 | -112.5999626 | 3612.10293 |
| 'out1cluster29' | 'linear' | 4989.394846 | 614.9513133 | 1.717592395 | 4055.720409 | 40.36461012 | -591.3942994 |
| 'out1cluster30' | 'linear' | 6.69E+02 | -6.29E+03 | 79.35865355 | 545.1821189 | -29.29807868 | -26539.92427 |
| 'out1cluster31' | 'linear' | -674.6507292 | -2769.303602 | -1032.677415 | -698.9050993 | -3.734626724 | 345943.2131 |
| 'out1cluster32' | 'linear' | -561.7966099 | -266.1074681 | -68.22000756 | 833.4946798 | 29.81730722 | 22814.62586 |
| 'out1cluster33' | 'linear' | 5518.879273 | -7039.186445 | -0.143105941 | 1016.540741 | -33.60652064 | 100.0382573 |
| 'out1cluster34' | 'linear' | -2800.678145 | 5794.09779 | 0.607938574 | 426.8467961 | -29.61921187 | -156.3173353 |
| 'out1cluster35' | 'linear' | -245.6517269 | 620.9568678 | -26.63234319 | -1707.944158 | -41.01251885 | 7920.920079 |
| 'out1cluster36' | 'linear' | -856.5774865 | -226.6093154 | -28.25042337 | 3738.447936 | 58.12459142 | 9090.172353 |
| 'out1cluster37' | 'linear' | 351.3881391 | -805.0174343 | -1.747814434 | -4565.469514 | -90.57588226 | 705.8169758 |
| 'out1cluster38' | 'linear' | -2754.422842 | -8454.279028 | -10.05344349 | 1076.285121 | -31.77218115 | 3319.48745 |
| 'out1cluster39' | 'linear' | -1005.850477 | 132.3269251 | -707.7740703 | 1423.432371 | 46.5144798 | 208723.1079 |
| 'out1cluster40' | 'linear' | 154.0287933 | 1409.495681 | 246.450245 | 11101.77985 | -24.73474829 | -80219.99556 |
| 'out1cluster41' | 'linear' | -7781.98483 | -4696.141667 | -0.131385895 | 1612.678884 | -32.93705439 | 88.79991164 |
| 'out1cluster42' | 'linear' | 1.21E+03 | -1.49E+04 | 54.05383316 | -619.2695555 | -51.94067082 | -16370.15312 |
| 'out1cluster43' | 'linear' | -5424.110118 | 2870.277232 | 260.8595116 | -493.2660476 | -33.03997477 | -84719.91314 |
| 'out1cluster44' | 'linear' | -852.6263988 | 913.0393774 | 35.94558272 | -1662.482576 | -31.85687416 | -10906.41196 |
| 'out1cluster45' | 'linear' | 3436.166242 | 1200.630046 | 48.13668902 | 15472.52509 | 396.0265961 | -15280.78397 |
| 'out1cluster46' | 'linear' | -1.23E+02 | -1.15E+03 | -7.038082978 | 1495.295431 | 24.41449307 | 2034.827639 |
| 'out1cluster47' | 'linear' | -1408.191819 | 1975.037561 | -23.62045479 | -4909.297619 | -133.4705337 | 7406.526225 |
| 'out1cluster48' | 'linear' | -360.7430935 | -387.2100684 | 94.45254222 | -4854.236232 | -159.3927849 | -28583.66642 |
| 'out1cluster49' | 'linear' | -20046.95481 | 3515.348688 | 5.197030441 | 3200.278684 | -51.66453887 | -1507.225551 |
| 'out1cluster50' | 'linear' | 491.8270597 | -321.6376264 | -1.005895867 | -4194.832381 | -89.10832415 | 464.5379373 |
| 'out1cluster51' | 'linear' | 2363.892115 | 3116.629517 | 8291.548538 | 1258.161581 | -38.61087385 | -2445985.235 |
| 'out1cluster52' | 'linear' | 481.1984302 | 259.8766674 | -73.91886015 | 2027.734756 | 38.37956906 | 22484.21489 |
| 'out1cluster53' | 'linear' | -1.83E+02 | 3.62E+03 | -4.263376939 | -17607.4431 | -279.0651181 | 1874.108574 |
| 'out1cluster54' | 'linear' | 765.973073 | -322.2504324 | -2.649868439 | -184.594985 | -32.10081786 | 907.7446439 |
| 'out1cluster55' | 'linear' | 4412.247107 | -1730.041689 | 134.9805004 | 365.4700068 | -29.15189532 | -41130.67271 |
| 'out1cluster56' | 'linear' | 623.4086975 | 14.05312752 | 62.04705257 | 88.3338946 | -18.91345495 | -20146.23287 |
| 'out1cluster57' | 'linear' | 6841.880223 | 4670.560605 | 20.08327254 | 1958.631059 | -28.76829629 | -6690.161628 |
| 'out1cluster58' | 'linear' | 6996.591986 | -2151.455348 | -23.1315406 | 336.4233628 | -33.47056842 | 7572.421918 |
| 'out1cluster59' | 'linear' | -6610.974027 | -2321.852043 | -15.73080718 | -10932.29443 | -89.5483179 | 4942.720219 |
| 'out1cluster60' | 'linear' | -1363.704165 | -3012.707769 | 503.4827522 | 5496.361841 | 93.61699083 | -148690.9552 |
| 'out1cluster61' | 'linear' | 331.2767338 | -1178.843376 | 533.678352 | 3346.529582 | 77.77086543 | -173559.2184 |
| 'out1cluster62' | 'linear' | 4882.317866 | 6122.880412 | -0.500652208 | -468.7646636 | -34.57625607 | 230.9130343 |
| 'out1cluster63' | 'linear' | -368.727872 | -194.8783324 | -1190.676924 | -9426.801363 | -77.63608179 | 363352.7418 |
| 'out1cluster64' | 'linear' | 35.73178201 | -443.9914242 | -8.360117707 | -5052.356162 | -116.0665827 | 2729.902036 |
| 'out1cluster65' | 'linear' | -3227.949346 | -5098.594331 | 81.61844709 | -9279.822956 | -86.61715788 | -23814.57693 |
| 'out1cluster66' | 'linear' | -5497.609091 | 713.80626 | 1.531492842 | 15905.36845 | 268.6415561 | -903.9891928 |
| 'out1cluster67' | 'linear' | -2879.805417 | 39537.45344 | 320.6577109 | 35835.10373 | 315.6495567 | -95309.77076 |
| 'out1cluster68' | 'linear' | 4804.535659 | -4503.942497 | 4.016885986 | 1431.349517 | -27.49775981 | -1310.450919 |
| 'out1cluster69' | 'linear' | -5823.950301 | -4100.924121 | -13.17301238 | 2126.503401 | -24.91164783 | 4440.885611 |
| 'out1cluster70' | 'linear' | -782.0566599 | 441.6315482 | -4.783399956 | 876.7319602 | 36.22785812 | 1506.835486 |
| 'out1cluster71' | 'linear' | -2959.982932 | 6488.290057 | -7.352576195 | 550.2963453 | -32.31818527 | 2440.040918 |
| 'out1cluster72' | 'linear' | -2963.010261 | -813.7007447 | -419.4307271 | -1410.155347 | -25.83977885 | 140558.78 |
| 'out1cluster73' | 'linear' | 17400.55253 | 3436.50904 | 345.4711155 | 1121.241563 | -50.36357066 | -105268.8671 |
| 'out1cluster74' | 'linear' | -7137.952141 | 55.30056873 | 68.82460235 | 6721.250346 | -30.33012382 | -22402.62336 |
| 'out1cluster75' | 'linear' | 6149.985368 | -1993.170566 | 106.8131375 | -9628.664418 | -83.46181583 | -31239.01217 |
| 'out1cluster76' | 'linear' | 8961.368164 | 1585.075045 | -52.28612761 | 2355.762519 | -30.4442155 | 17028.49101 |
| 'out1cluster77' | 'linear' | 667.1740162 | 302.3528335 | -8.915792214 | 3841.326738 | 94.60824512 | 2575.487717 |
| 'out1cluster78' | 'linear' | -1367.329911 | 12510.24097 | 0.042830122 | -2345.924633 | -58.57390012 | 123.9182469 |
| 'out1cluster79' | 'linear' | -770.061847 | 2431.677527 | 113.1319678 | -11298.13637 | -91.48230919 | -33085.8991 |
| 'out1cluster80' | 'linear' | 2407.612424 | -10823.22688 | -2.853628816 | 1293.874393 | -34.13431302 | 988.0745 |
| 'out1cluster81' | 'linear' | -1568.341327 | -4204.249111 | 27.55681626 | -2366.712238 | -79.71269974 | -8838.471139 |
| 'out1cluster82' | 'linear' | 2349.710454 | -6818.348794 | 34.10416979 | -8499.131791 | -80.47871966 | -9810.214933 |
| 'out1cluster83' | 'linear' | 25884.86007 | 8981.752276 | 46.30755427 | -263.6663012 | -72.54030076 | -13479.0743 |
| 'out1cluster84' | 'linear' | 3025.916221 | -765.1826863 | -0.30673893 | 2724.104404 | -23.70677156 | 104.1986361 |
| 'out1cluster85' | 'linear' | -12740.79089 | -5449.795795 | 30.52993131 | -952.4617408 | -54.64287955 | -9193.584527 |
| 'out1cluster86' | 'linear' | 2626.636523 | -230.1834011 | -0.910397492 | -2945.384318 | -104.2198823 | 424.599338 |
| 'out1cluster87' | 'linear' | 117.7712066 | -502.4717369 | -1.18855018 | 2869.699097 | 76.71489431 | 232.8505693 |
| 'out1cluster88' | 'linear' | -2726.808068 | -4922.46417 | 70.48353786 | 708.8915487 | -28.11455431 | -23572.79408 |
| 'out1cluster89' | 'linear' | 8378.754288 | -12764.42183 | 15.04936275 | -1744.557756 | -57.87414072 | -4450.47825 |
| 'out1cluster90' | 'linear' | -177.079031 | -2000.880259 | -9.486689778 | 3814.411081 | 60.79656608 | 2792.191376 |
| 'out1cluster91' | 'linear' | -3758.941041 | -3065.635556 | 1487.526711 | -1304.911052 | -22.26846154 | -453674.7008 |
| 'out1cluster92' | 'linear' | -86.92524721 | 720.1692043 | -44.28243806 | 529.4014605 | -6.589808051 | 13064.14549 |
| 'out1cluster93' | 'linear' | 887.7535475 | 1091.815019 | -400.2633893 | 1098.236161 | -21.73258437 | 122073.5811 |
